# Supplementary material for: Development and Effects of Leukemia Nursing Simulation Based on Clinical Reasoning
Source: Int J Environ Res Public Health. 2021 Apr 15;18(8):4190. doi: 10.3390/ijerph18084190 (PMC8071219; doi:10.3390/ijerph18084190)
Supplement: Supplementary file 1 [file ijerph-18-04190-s001.zip › ijerph-1168652-SI.pdf]

Table S1. Overview of nursing simulation about acute myelocytic leukemia (AML).

Learning Objectives	
1. Collect information related to the patient's symptoms.<br>2. Conduct clinical reasoning based on assessment data.<br>3. Apply nursing according to priority based on clinical reasoning.<br>4. Evaluate patient response to the nursing provided.	
**Connectivity with Nursing Skill**	- Measurement of oxygen saturation and application of ECG monitor,<br>- Oxygen therapy using nasal cannula
**Scenario Overview**	In the developed scenario, a 70-year-old female patient with AML was hospitalized in the hematology/oncology unit for a second chemotherapy cycle. The patient had a chemo port on the right upper chest. She complained of general weakness and weight loss, and the pharmacy sent chemotherapy medication on that day. The patient also experienced discomfort due to oral bleeding. The patient's log sheet, including her general doctor's prescription, was provided beforehand. You are the nurse in charge of the day shift patient, admitted to the hematological oncology ward through an outpatient for the 2nd chemotherapy yesterday. After taking over for a nurse from the night shift, you enter the hospital room to care for the patient.
**Role**	**Student**<br>Primary nurse, Secondary nurse, Observers/Recorder, Nurse Practitioner<br>**Instructor**<br>- Answering nurse's questions as a patient<br>- Questions and sound effects according to the patient's condition<br>- The role of a doctor who reports the patient's condition over the phone and makes prescriptions<br>- Role of the laboratory to show the blood test results in the laboratory or X-ray results<br>- Giving cues so students' interventions and skills can be completed within time.<br>- Do not give possible answers but wait so you can think for yourself.
